# Effects of lumbar stabilization exercises on serum biomarker levels and clinical outcomes in lumbar disc herniation: a subgroup analysis of a randomized controlled trial

**DOI:** 10.55730/1300-0144.6004

**Published:** 2025-05-07

**Authors:** Birgül MORKOÇ, Onur AKTAN, Gamze SÖNMEZ, Ebru BODUR, Jale KARAKAYA, Bayram KAYMAK, Sevil BİLGİN

**Affiliations:** 1 Department of Physical Medicine and Rehabilitation, Hacettepe University, Ankara, Turkiye; 2 Department of Medical Biochemistry, Hacettepe University, Ankara, Turkiye; 3 Department of Biostatistics, Hacettepe University, Ankara, Turkiye; 4 Faculty of Physical Therapy and Rehabilitation, Hacettepe University, Ankara, Turkiye

**Keywords:** Intervertebral disc herniation, cytokines, physical therapy, exercise, beta-endorphin, endocannabinoids

## Abstract

**Background/aim:**

The aim of our study was to investigate the effectiveness of lumbar stabilization exercises on serum biomarker levels and clinical outcomes in lumbar disc herniation (LDH) patients with and without neurological deficit.

**Materials and methods:**

40 patients with neurological deficits (WND) and 34 patients without neurological deficits (WOND) diagnosed with LDH were included in this study. The patients’ WND and WOND were randomized into stabilization exercises (SE) and general exercises (GE) groups. Stabilization exercises or general exercises were applied for six weeks. Serum levels of tumor necrosis factor alpha (TNF-α), interleukin (IL)-1β, IL-6, IL-4, beta-endorphin (BE), anandamide (AEA), and 2-arachidonoylglycerol (2-AG) were measured using the enzyme-linked immunosorbent assay. All patients filled out the Visual Analog Scale, Oswestry Disability Index, Beck Depression Inventory, and Beck Anxiety Inventory.

**Results:**

In patients WND and WOND, SE did not show any statistically significant difference in relation to TNF-α, IL-1β, IL-6, IL-4, BE, AEA, and 2-AG (p > 0.05). There were no statistically significant differences between the SE and GE groups in pain intensity and disability outcomes in patients WND (p > 0.05). SE group showed greater reductions in depression and anxiety scores compared to the GE group (p < 0.05). In patients WOND, there were no differences in pain, disability, or depression results between the SE and GE groups (p>0.05), whereas the decrease in anxiety score was greater in the SE group (p < 0.05).

**Conclusion:**

Our results demonstrate that no effect of lumbar stabilization exercise is seen on circulating levels of TNF-α, IL-1β, IL-6, IL-4, BE, AEA, and 2-AG. Further exercise trials are needed to investigate what type, duration, and intensity of exercise is relevant for biomarkers that may play a role in the immune process.

## 1. Introduction

Lumbar disc herniation is one of the most common causes of low back pain, affecting about 1–3% of the total population annually, mostly people between 30 and 50 years of age. LDH has a serious impact on people’s daily and professional lives, and its rising incidence is causing an enormous burden on society [1]. When symptomatic, it usually manifests as low back pain and pain radiating down the leg. Severe cases are accompanied by sensory, motor, and autonomic deficits [2]. Neurological deficits are the most feared complications of LDH and can lead to long-term disability in a small proportion of patients [3].

Low back pain associated with LDH is not merely a mechanical problem; it also involves complex neuroinflammatory and biochemical mechanisms that lead to chronic pain and neurological dysfunction. Current literature emphasizes the role of proinflammatory cytokines such as TNF-α, IL-1β and IL-6 in the pathophysiology of LDH, indicating that pain and neurological symptoms are closely related to systemic inflammation and neuroimmune responses [4, 5]. These inflammatory responses contribute to the persistence of pain, neural sensitization, and delayed healing. Insufficient activation of antiinflammatory mechanisms further complicates this process. In this context, approaches that target not only mechanical dysfunctions but also inflammatory markers are becoming increasingly important.

Tumor necrosis factor alpha (TNF-α), interleukin 1 beta (IL-1β), and IL-6 are proinflammatory cytokines. TNF-α is a pleiotropic cytokine that can stimulate inflammatory responses affecting the synapses and myelin sheath, exerts a cytotoxic effect that supports apoptosis, and causes nerve swelling and neuropathic pain [6]. IL-1β increases neuronal excitability, activates monocyte/macrophages, and may exacerbate inflammatory cell infiltration [7]. IL-6 is a strong marker of pain in discogenic low back pain. It can induce the recruitment of inflammatory cells, activate the release of inflammatory mediators, and trigger degeneration of the intervertebral discs. Levels of these cytokines have been shown to be higher in individuals with disc herniation compared to healthy individuals [8, 9]. In contrast, IL-4 is an antiinflammatory cytokine known to inhibit proinflammatory cytokines such as TNF-α, IL-1β, and IL-6. It can stimulate B-cell activation and T-cell differentiation and suppress macrophage activation [10]. In addition to cytokines, beta-endorphins (BEs) and the endocannabinoid system are known to play a role in the inflammatory process. BEs are potential biomarkers in patients with chronic pain, with elevation suggested to be associated with a decrease in maximum pain intensity [11, 12]. Anandamide (AEA) and 2-arachidonoylglycerol (2-AG) are the main endocannabinoids and are produced from cell lipid membrane precursors. The endocannabinoid system regulates pain control via the activity of cannabinoid receptors CB1 and CB2. CB1 inhibits nociceptive transmission to the thalamus at the supraspinal level, while CB2 is more involved in regulating immune responses in the spinal cord associated with chronic pain [13–17].

Exercise has been shown to have the potential to regulate these immune responses in low back pain [18–20]. McKenzie exercises were reported to increase TNF-α production but not affect IL-6 levels in acute low back pain [21]. In chronic nonspecific low back pain, a 4-week virtual reality exercise program was found to cause a greater decrease in TNF-α and IL-6 levels and a greater increase in IL-4 levels compared to isokinetic and conventional exercises [22]. Molina et al. showed that in individuals with nonspecific low back pain, TNF-α levels increased and IL-6 levels decreased in the group that performed traditional core strengthening exercises, whereas those who performed specific stabilization exercises showed increased IL-6 levels and decreased TNF-α levels [18]. In a study examining the immediate effect of lumbar stabilization exercises on BE levels, Paungmali et al. reported that serum BE levels were higher 1 minute after stabilization exercises compared to 1 minute before exercise [19].

Stabilization exercises are theoretically defined as an approach that targets the deep muscle groups responsible for providing segmental control of the spine, aiming to retrain motor control mechanisms [23]. Clinically, these exercises have been shown to contribute significantly to pain reduction, improved functional capacity, enhanced quality of life, and the prevention of recurrences [24–27]. There is a growing interest in the notion that these exercises may exert effects beyond mechanical factors, potentially influencing inflammatory responses and biological markers. Moreover, it is suggested that patients’ responses to exercise may vary depending on their neurological deficit status. In recent years, the effects of exercise on physiological processes such as inflammation, tissue healing, and oxidative stress have begun to be objectively evaluated through biomarkers [28]. Nevertheless, studies investigating these biological responses in individuals with low back pain remain limited. Targeting both symptom control and underlying mechanisms is essential in the management of low back pain. This approach not only enhances our understanding of the biological basis of exercise-based rehabilitation, but also supports the development of more individualized, effective, safe, and goal-oriented treatment strategies grounded in scientific evidence.

The inflammatory process is implicated in the pathogenesis of LDH. Our aim in this study was to investigate whether exercise triggers immune responses in LDH. We hypothesized that stabilization exercises may involve an immune component that supports its pain-reducing effect. Our secondary aim was to support the evidence of the positive effect of stabilization exercises on pain, disability, and emotional outcomes reported in the literature. Therefore, we investigated the effects of a 6-week stabilization exercise intervention on TNF-α, IL-1β, IL-6, IL-4, BE, AEA, and 2-AG levels and clinical outcomes in LDH patients with and without neurological deficits. This study aims not only to address the gap in the existing literature but also to contribute to the advancement of clinical practices by incorporating mechanical and biochemical factors underlying pain and dysfunction. The findings obtained may offer a more holistic understanding of treatment by revealing not only the symptomatic outcomes but also the physiological and molecular effects of stabilization exercises.

## 2. Materials and methods

### 2.1. Study design and participants

A total of 74 individuals with LDH (44 female, 30 male) were included in this randomized controlled study (NCT04912388, ClinicalTrials.gov). Clinical assessments and exercise interventions were performed by an experienced physiotherapist that was not blinded. Biochemical analyses were carried out by experienced biochemists who were blinded. The participants were also blinded to their assigned intervention.

Individuals 20–55 years of age who presented to the Department of Physical Medicine and Rehabilitation of Hacettepe University Hospitals with low back pain, were diagnosed with LDH by MRI, and had pain severity ≥3 on the visual analog scale (VAS) were included in the study. The LDH patients were divided into two groups, those with neurological deficit (WND) and those without neurological deficit (WOND). Individuals were included in the WND group if they had at least one of the following: positive straight leg raise test, loss of sensation, reflex abnormality, and motor weakness. The WND and WOND patients were randomly assigned to receive the stabilization exercises (SE) and general exercise (GE) interventions using the sealed envelope method.

Patients with any of the following were excluded: surgical treatment for LDH, lumbar scoliosis, lumbar vertebral fracture, generalized musculoskeletal pain, systemic or inflammatory disease, any allergic, neurological, or psychiatric disease, malignancy, alcohol or drug use, pregnancy, and breastfeeding.

### 2.2. Descriptive and clinical measures

The participants’ demographic data (age, sex, height, weight, body mass index [BMI], occupation, smoking status), disease-related information (level and type of herniation), and other clinical information (pain duration, medication use) were recorded. Occupations were classified as involving strenuous work (lifting or carrying heavy objects, forward bending), moderate work (household chores), and mild work (sitting and constant posture) [29]. At baseline and 6 weeks, the LDH patients were evaluated in terms of pain using the VAS [30], disability using the Oswestry Disability Index [31], and emotional state using the Beck Depression Inventory [32] and Beck Anxiety Inventory [33].

### 2.3. Biochemical analysis

Serum levels of TNF-α, IL-1β, IL-6, IL-4, BE, AEA, and 2-AG were measured at baseline (before the intervention) and at week 6 (after the intervention). Venous blood samples were collected into two 5-mL biochemistry tubes which were centrifuged at 3000 rpm for 10 min. The isolated serum samples were stored at −30 °C until analysis using enzyme-linked immunosorbent assay (ELISA) kits (Reed Biotech).

### 2.4. Intervention

The patients participated in their assigned exercise program for 3 sessions per week for 6 weeks. In addition to physiotherapy modalities consisting of hot pack, ultrasound, transcutaneous electrical nerve stimulation (TENS), and traction, participants in the SE group performed stabilization exercises, and those in the GE group performed general exercises. Within the physiotherapy program, patients underwent superficial heat therapy using a hotpack maintained at 40 °C for 20 min. TENS device set to a frequency range of 60–120 Hz with a pulse duration of 50–100 ms for 20 min. This is followed by ultrasound treatment applied for 6 min, utilizing a frequency of 1 MHz and intensity of 1.2 W/cm^2^. The session concluded with lumbar traction therapy, applied at a force equivalent to 50% of the patient’s body weight for 10 min, with the traction weight increased by 1–2 kg daily based on the patient’s tolerance. Each exercise was practiced 10 times under the supervision of a physiotherapist. The sessions lasted a total of 45 min, including 5 min of warm-up and 5 min of cool-down.

### 2.5. Stabilization exercises

Stabilization exercises started with abdominal hollowing (pulling the abdomen up and in), which provides cocontraction of the deep muscles. Patients were placed in the supine hook position and taught diaphragmatic breathing. At the end of the expiration, they were asked to pull their abdomen up or inward or to contract the muscles of the pelvic region. The accuracy of the abdominal hollowing maneuver was checked with a stabilizer placed in the lumbar region. The patients were asked to continue the contraction for 30–45 s while continuing to breathe. In the first week, the patients learned the abdominal hollowing maneuver and maintained it during unilateral extremity movements in the supine position. Starting in the second week, the movements progressed through crawling, sitting, and standing positions and the support surface was reduced. The accuracy and continuity of the abdominal hollowing maneuver were checked in each position. Bilateral, contralateral, ipsilateral, and reciprocal extremity movements were added to the program to improve dynamic stabilization. Resistance training was started from the third week using dumbbells, sandbags, and resistance bands as auxiliary equipment. In the fourth week, the patients transitioned from stable surfaces to unstable surfaces using exercise balls. In the fourth and fifth weeks, the patients progressed towards exercises targeting global muscle activity. Each exercise was performed 10 times. Each session of the SE program started with a warm-up and ended with a cool-down involving hamstring, hip flexor, lumbar extensor, and piriformis muscle stretching exercises. The hamstring stretching exercise was started after the third week for patients in the WND group with a positive straight leg raise test (Appendix, Table 1).

### 2.6. General exercises

Patients in the GE group received a program consisting of stretching exercises and strengthening exercises for the core and hip muscles (Appendix, Table 2). Stretching exercises targeted the hip flexor, lumbar extensor, piriformis, and hamstring muscles. These exercises were used for warm-up and cool-down purposes in all sessions. The hamstring stretching exercise was started after the third week for patients in the WND group with a positive straight leg raise test. In addition to stretching exercises, posterior pelvic tilt, abdominal crunch, back extension, isometric gluteus maximus, hip abduction, hip extension, and hip adduction exercises were prescribed. The exercises were performed under the supervision of a physiotherapist for 6 weeks.

### 2.7. Statistical analysis

Statistical analyses were performed in IBM SPSS Statistics version 23.0. The normality of data distributions was evaluated with the Shapiro-Wilk test. As descriptive statistics, mean and standard deviation or median and range were given for numerical variables and number and percentage were given for qualitative variables. Mann-Whitney U test was used to compare two independent groups in terms of numerical variables, and Pearson’s chi-square test or Fisher-Freeman-Halton exact test was used to compare in terms of qualitative variables. Comparisons of numerical variables were made with the Wilcoxon test. Significance was accepted at p < 0.05. The sample size was calculated using the program G*Power 3.1.9.2 to test the significance between and within groups. Minimum sample size was calculated with a power of 80% and a 5% alpha error and was estimated as 37 patients for each group, and a total of 74 patients [4].

## 3. Results

Of 79 people with LDH assessed, 2 declined to participate, and 3 did not meet the inclusion criteria. Therefore, 74 patients were included in the study between February 2022 and February 2024. The flow chart of the study is shown in Figure. The demographic and clinical characteristics of the participants are given in Table 3. The WND and WOND groups were similar in terms of age, sex, BMI, smoking, duration of pain, and type and duration of herniation. None of the patients had used medication since being diagnosed with LDH.

Descriptive information about the neurological deficits of WND patients in the SE and GE groups is shown in Table 4.

The median serum biomarker levels of the WND and WOND patients before and after the interventions are shown in Table 5. The differences between pre and postintervention values were not significant within the SE and GE groups and did not differ significantly between the groups among both WND and WOND patients (p > 0.05).

Pre and postintervention pain severity, disability, depression, and anxiety scores are compared within and between the SE and GE groups in Table 6. Both the SE and GE interventions were associated with significant decreases in pain severity, disability, and depression and anxiety scores in WOND patients (p < 0.05). Among WND patients, pain, disability, and depression scores decreased significantly in both the SE and GE groups, while anxiety score decreases significantly only in the SE group (p < 0.05). The postintervention changes in pain severity, disability, and depression values did not differ significantly between the SE and GE groups among WOND patients (p > 0.05), whereas anxiety values showed a statistically greater decrease in the SE group compared to the GE group (p < 0.05). Among WND patients, both depression and anxiety scores decreased significantly more in the SE group than in the GE group (p < 0.05), while there was no statistically significant difference in the reductions in pain and disability between the SE and GE groups (p > 0.05).

## 4. Discussion

The present study evaluated the impact of exercise on biomarker levels and clinical assessment results in individuals with LDH with and without neurological deficits. Our findings showed that there was no significant change in serum TNF-α, IL-1β, IL-6, IL-4, BE, AEA, or 2-AG levels with stabilization exercises. However, stabilization exercises were associated with greater decreases in depression and anxiety levels among LDH patients with neurological deficit and a greater decrease in anxiety level among LDH patients without neurological deficit when compared with general exercises.

Studies examining the effects of lumbar stabilization exercises on pro and antiinflammatory cytokines remain limited. Oghumu et al. found a significant increase in IL-6 levels after 10 weeks of lumbar stabilization exercises in individuals with nonspecific chronic low back pain [34]. Similarly, Molina et al. reported that in individuals with nonspecific low back pain, the group performing traditional trunk strengthening exercises showed increased TNF-α levels and decreased IL-6 levels. In contrast, specific stabilization exercises increased IL-6 levels and decreased TNF-α levels [18]. Nambi et al. also reported that isokinetic training reduced inflammation, as evidenced by changes in TNF-α, IL-6 and IL-4 levels compared to those in other study groups. In their comparison of isokinetic and stabilization exercises, they concluded that the use of a Swiss ball in the stabilization exercise group allowed the nervous system to undergo neuroplastic changes, potentially altering the inflammatory process [35].

The fact that similar changes were not observed in our study may be explained by factors such as differences in the intensity and duration of the exercise protocol. Randomized controlled exercise studies have demonstrated that aerobic exercise performed in individuals with high levels of inflammation is effective in reducing proinflammatory biomarker levels. Petersen et al. found that IL-6 levels increased 20-fold after 90 min of treadmill running at 75% of maximum oxygen consumption, and increased by 100-fold after running a marathon [36]. It is possible that we were unable to demonstrate changes in IL-6 levels because the stabilization exercises employed in our study were of light to moderate intensity. Nambi et al. applied virtual reality and isokinetic exercises to soccer players with chronic nonspecific low back pain and observed decreased TNF-α and IL-6 levels, along with increased IL-4 levels [22]. It has been reported that individuals enjoy virtual reality training, progress to the next level quickly, and expend more energy than other exercises. This type of training typically involves high-frequency activities that may changes in pro and antiinflammatory cytokines. In isokinetic training, it has been noted that exercises performed at various angular velocities with maximum rotational force can influence proinflammatory cytokine levels. Similarly, Faelli et al. found a significant decrease in IL-1β levels following 24 sessions of resisted exercise training [37]. Regular exercise has also been shown to reduce IL-1β levels and increase IL-10 levels. Therefore, the low intensity and limited energy expenditure associated with stabilization exercises may have influence the outcomes of our study.

Long-term exercise involving a significant muscle mass is necessary to elicit a marked systemic IL-6 response. Among various factors, exercise duration is considered the most important factor determining the magnitude of the systemic IL-6 response. Therefore, short-term or low-intensity exercise is unlikely to induce systemic IL-6 effects to the extent that might be expected [38]. In the exercise program used in our study, the sessions lasted less than 1 h. Another reason IL-6 levels may not have changed is that IL-6 possesses both proinflammatory and antiinflammatory properties [21]. Al-Obaidi and Mahmoud demonstrated that TNF-alfa production increased after four weeks of McKenzie exercises in individuals with acute low back pain, whereas IL-6 levels remained unchanged. They attributed the lack of change in IL-6 levels to its dual role as both a pro and antiinflammatory cytokine [21].

Plasma BE levels are also reported to increase during exercise, especially when exercise intensity reaches the anaerobic level [39]. A certain level of metabolic and thermal stress is required during exercise for BE release to occur [40]. It is not clear whether lumbar stabilization exercises create sufficient physical stress to induce BE release and increase plasma BE levels in individuals with chronic low back pain [41]. Only a single study was identified that examined the effects of lumbar stabilization exercises on BE levels. In a study examining the immediate effect of lumbar stabilization exercises on BE levels, Paungmali et al. reported that serum BE levels were higher 1 min after stabilization exercises compared to 1 min before exercise [19]. They suggested that lumbar stabilization exercises may affect circulating BE levels and that this may explain one of the possible aspects of pain mechanisms based on endogenous analgesic peptides. Since this study examines the acute effects of exercise, it is not possible to make a meaningful comparison with the results of our study. Studies on the effects of exercise on BE release are controversial. Goldfarb et al. showed that very intense exercise (80% VO_2_max) led to significant increases in BE concentrations, which occurred early in exercise (within first 5–10 min) [42]. However, no significant changes were observed in studies with less intensive protocols (80%-85% maximum heart rate) [43, 44]. In another study, it was concluded that there was no significant change in BE concentration during low- to moderate-intensity exercise [45]. The differences between the results of these studies may be due to the differences in the exercise dosage used in the experimental protocols. In our study, we observed no statistically significant difference in BE levels after stabilization exercises or general exercise in individuals with LDH with and without neurological deficit. This may be due to the low exercise intensity and the fact that biochemical analyses were not performed immediately after exercise. In our study, there was a clinically significant but statistically nonsignificant increase in BE levels with stabilization exercises, suggesting that these exercises provide a sufficient stimulus for BE release that may cause a clinical increase. The classic function of BE is analgesic, with BE shown to be 20 to 33 times more potent than morphine. Increasing stabilization exercise intensity to at least 70% of the maximum oxygen consumption may result in increased BE levels and allow the analgesic effect of stabilization exercises to be objectively demonstrated.

The first study to reveal the effect of physical activity on endocannabinoids in humans was conducted by Sparling et al. who showed that AEA levels increased and 2-AG did not change in healthy individuals after 50 min on a treadmill or stationary bicycle at 70%-80% of maximum heart rate [46]. Marin Bosch et al. determined that AEA concentrations were higher after exercise at 80% of maximum heart rate than after exercise at 70% of maximum heart rate [47]. Many authors have reported that moderate-intensity exercise is associated with greater increases in AEA. However, Cedernaes et al. showed that AEA levels remained constant and 2-AG levels increased after 30 min on a cycle ergometer [48]. Exercise history is also a factor that can change endocannabinoid responses. It has been demonstrated that more physically active individuals may show a stronger AEA response than those with low physical activity. Endocannabinoid levels may also be affected by factors such as fasting status, the time and content of the last meal, BMI, sex, previous health problems, sleep, and the menstrual cycle [49–52]. The lack of significant changes in endocannabinoid levels in our study may be attributed to the effect of these factors.

Physical exercise is the primary physiotherapeutic strategy for low back pain, yet there is insufficient evidence to show that a particular exercise is optimal for any one person. Although the mechanical effects of lumbar stabilization exercises in individuals with low back pain are well known, their biochemical effects have not been fully elucidated. More studies are needed to reveal clinically significant changes and improve our understanding of the role of exercise type, duration, and intensity in the relationship between low back pain and inflammation. Given the far-reaching detrimental effects of the inflammatory process, it is critical to identify treatments that reduce inflammation. However, there has been little evidence to date of therapies that can effectively lower elevated inflammatory biomarkers. Studies using different biochemical analysis methods, different exercise durations and intensities, and obtaining measurements at the onset of back pain and at specific time periods in larger samples of LDH patients will provide more detailed insight into the effect of exercise on biochemical factors on the pro and antiinflammatory processes.

In the present study, we determined that both stabilization exercises and general exercise were effective in reducing pain and disability in individuals with and without neurological deficits, but neither was superior. This may be due to the fact that both stabilization exercises and general exercises were well structured, and the patients performed their exercises under the supervision of a physiotherapist. There are studies in the literature supporting the results of our study. In their study on patients with chronic non-specific low back pain, Tøndel et al. showed that stabilization exercises were not superior to general exercises in terms of pain and disability results [53]. Shamsi et al. also reported that both exercise programs were equally effective in improving pain and disability [54]. Contrary to our results, there are also studies in the literature stating that stabilization exercises are more effective against pain and disability. Akodu and Akindutire showed that stabilization exercises were more effective in improving pain and disability and attributed this improvement to the restoration of normal control of the multifidus and transversus abdominis, local muscles that stabilize the spine [55]. Wang et al. observed that stabilization exercises were more effective in reducing pain and disability in short-term follow-up, but there was no significant difference between the groups in measurements made at 6 and 12 months [56].

In our study, we found that stabilization exercises were more effective than general exercise in reducing depression and anxiety in individuals with LDH with neurological deficits and in reducing anxiety in individuals with LDH without neurological deficits. Stabilization exercises are a complicated and progressive program performed by adding resistance in different positions and surfaces. This program may have allowed patients to realize that movement is not a real threat, thereby reducing their fear of movement. Exercise is known as a form of distraction that diverts attention from one’s thoughts. Moderate-intensity stabilization exercises may have prevented patients from over-thinking and helped them escape distressing thoughts. Akodu and Akindutire applied an 8-week stabilization exercise program in individuals with chronic nonspecific low back pain and showed that depression and anxiety levels improved [55]. They attributed the improvement in emotional state to the decrease in pain-related disability.

There are theories in the literature that hormonal changes may have an effect on emotional state [57]. In our study, it was observed that BE levels were higher after stabilization exercises in LDH patients with and without neurological deficits. Based on this theory, we think that elevated BE levels may result in improvement in emotional state.

This study has some limitations. The first may be our biochemical analysis method. In our study, biochemical factors were analyzed from venous blood samples. Using methods that can analyze cytokine levels locally, such as the microdialysis method, may yield different results. Another possible limitation is the timing of blood sampling. Blood samples were obtained after 6 weeks of treatment. The effectiveness of exercise may have been demonstrated more clearly by performing biochemical analyses at different times, such as immediately before, during, and immediately after exercise.

## 5. Conclusion

In patients with LDH, stabilization exercises may not be an effective way to significantly influence circulating levels of TNF-α, IL-1β, IL-6, IL-4, BE, AEA, and 2-AG. Further exercise trials are needed to investigate what type, duration, and intensity of exercise is relevant for biomarkers that may play a role in the immune process. Stabilization and general exercises appear to improve or cure pain and functional level similarly. Stabilization exercises are also more effective in improving emotional state in LDH patients with and without neurological deficits. Therefore, stabilization exercises should be considered a favorable method for treating clinical symptoms in patients with LDH.

## Supplementary Information



## Figures and Tables

**Figure:**
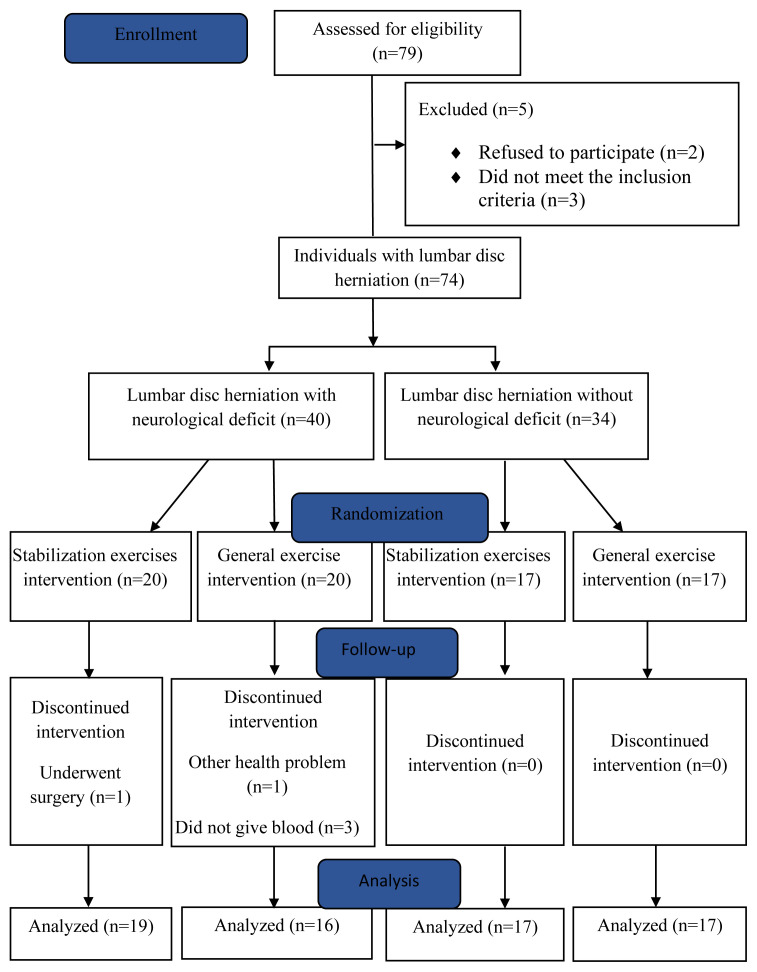
Flowchart of the study.

**Table 1:** Stabilization exercise protocol.

Week	Exercises	Week	Exercises
1	Abdominal hallowing	4	One-leg shoulder bridge
	Unilateral shoulder flexion exercise		Lower body rotation to the right in table top
	Unilateral hip flexion exercise		Lower body rotation to the left in table top
	G. Maximus isometric		Plank
	Ipsilateral shoulder and hip flexion		Hip extension
	Clam shell exercise		Unilateral shoulder flexion
			Contralateral shoulder and hip flexion
			Resisted shoulder flexion
			Resisted hip extension
			Swiss ball crunches
			Abdominal crunch in table top
2	Contralateral shoulder and hip flexion	5	Stability ball bird dog
	Quadruped unilateral hip extension		Oblique curl up to the left in table top
	Quadruped unilateral shoulder extension		Oblique curl up to the right in table top
	Clam shell exercise with horizontal adduction		One-arm and one-leg shoulder bridge
	Contralateral shoulder and hip flexion in sitting		Oblique curl up to the right with lower extremity movements
	Hip extension in prone		Oblique curl up to the right with lower extremity movements
	Shoulder and hip extension in prone		Contralateral shoulder and hip flexion in standing
	Back extension		Bird dog with a swiss ball
	Quadruped hip extension		
3	Bear plank	6	Superman
	Bird dog		Reverse plank
	Resisted bilateral shoulder flexion		Side plank
	Resisted ipsilateral shoulder and hip flexion		Lunge
	Hip abduction in side lying		Squat
	Bridge		Back extension
	Reciprocal hip flexion extension		
	Kneeling sit and stand exercise		

**Table 2:** General exercise protocol.

Exercises
Stretching exercises (Hamstring, hip flexor, lumbar extensor, piriformis muscles)
Posterior pelvic tilt
Abdominal crunch
Back extension
G. Maximus isometric
Hip abduction
Hip extension
Hip adduction

**Table 3:** Demographic, medical and clinical characteristics of groups.

	WOND (n = 34)			WND (n = 40)		
	SE	GE	p	SE	GE	p
Age, mean (SD), years	41.29 (9.33)	46.12 (6.40)	.09^a^	41 (10.01)	45.25 (6.68)	.13^a^
Gender						
Male	5 (29.4)	9 (52.9)	.16^b^	5 (25)	11 (55)	.05^b^
Female	12 (70.6)	8 (47.1)		15 (75)	9 (45)	
BMI, mean (SD), kg/m^2^						
Normal	9 (52.9)	4 (23.5)	.09^c^	11 (55.0)	5 (25.0)	.16^c^
Overweight	8 (47.1)	10 (58.8)		6 (30.0)	9 (45.0)	
Obese	-	3 (17.6)		3 (15.0)	6 (30.0)	
Occupation (n/%)						
Light work	1 (5.9)	-	.03^c^	2 (10.0)	-	.13^c^
Medium work	14 (82.4)	8 (47.1)		12 (60.0)	9 (45.0)	
Heavy work	2 (11.8)	9 (52.9)		6 (30.0)	11 (55.0)	
Smoking, yes	8 (47.1)	12 (70.6)	.16^b^	8 (40.0)	11 (55.0)	.34^b^
Pain duration, month						
3–6	6 (35.3)	4 (23.5)	.60^c^	9 (45.0)	5 (25.0)	.37^c^
6–12	3 (17.6)	1 (5.9)		3 (15.0)	6 (30.0)	
12–18	2 (11.8)	2 (11.8)		-	-	
18–24	6 (35.3)	10 (58.8)		8 (40.0)	9 (45.0)	
Disc herniation on magnetic resonance imaging						
Level						
L1/2	-	-	.27^c^	-	1 (5.0)	.81^c^
L2/3	-	2 (11.8)		1 (5.0)	1 (5.0)	
L3/4	-	-		2 (10.0)	-	
L4/5	6 (35.3)	8 (47.1)		6 (30.0)	6 (30.0)	
L5/S1	11 (64.7)	7 (41.2)		11 (55.0)	12 (60.0)	
Type						
Protrusion	17 (100)	15 (88.2)	.49^c^	10 (50.0)	17 (85.0)	.05^c^
Extrusion	-	2 (11.8)		8 (40.0)	3 (15.0)	
Sequestration	-	-		2 (10.0)	-	

WOND: Lumbar disc herniation patients without neurological deficit; WND: Lumbar disc herniation patients with neurological deficit; BMI: Body mass index; SE: Stabilization exercises group; GE: General exercise group. Values are presented as a number (percentage) of participants unless otherwise indicated.

a Independent-sample t test,

b Pearson Chi-Square test,

c Fisher-Freeman-Halton exact test.

**Table 4:** Types of neurological deficits in herniated disc patients with neurological deficits.

Type of neurological deficit (n)	SE	GE
Sensory	6	4
Motor	10	11
Reflex	4	1
SLR	15	11

n, Number of participants; SE, Stabilization exercises group; GE, General exercise group; SLR, Straight leg raise test.

**Table 5:** The descriptive and statistical comparisons of the biochemical markers.

		WOND			WND		
		SE (n = 17)	GE (n = 17)	p	SE (n = 19)	GE (n = 16)	p
TNF-α	Pre	4.57 (2.44–6.16)	4.96 (3.37–17.01)	.95^b^	5.18 (2.61–10.00)	4.70 (3.60–9.76)	.46^b^
	Post	4.43 (2.60–7.09)	4.81 (3.52–5.63)		5.20 (2.75–7.98)	4.99 (3.73–7.87)	
	p	.44^a^	.50^a^		.49^a^	.84^a^	
IL-1β	Pre	2.03 (0.49–6.01)	2.59 (0.47–18.97)	.61^b^	2.71 (0.55–21.17)	2.38 (0.48–9.76)	.64^b^
	Post	2.25 (0.48–7.09)	2.54 (0.49–22.75)		2.87 (0.52–14.21)	2.87 (0.47–7.87)	
	p	1.00^a^	.59^a^		.90^a^	.33^a^	
IL-6	Pre	0.65 (0.26–1.67)	0.62 (0.43–1.87)	.87^b^	0.50 (0.18–0.72)	0.54 (0.24–1.21)	.71^b^
	Post	0.66 (0.37–1.16)	0.66 (0.40–1.74)		0.51 (0.28–1.14)	0.62 (0.23–2.11)	
	p	.30^a^	.35^a^		.34^a^	.47^a^	
IL-4	Pre	10.05 (3.70–25.86)	11.96 (5.45–25.23)	.92^b^	13.25 (5.83–22.37)	12.14 (3.70–31.83)	.78^b^
	Post	10.32 (4.34–21.90)	11.47 (4.88–34.60)		13.66 (5.22–23.97)	12.71 (4.34–36.76)	
	p	.72^a^	.72^a^		.42^a^	.38^a^	
BE	Pre	991.27 (528.43–2431.74)	996.55 (168.24–1566.43)	.68^b^	1002.58 (52.39–3199.62)	984.71 (151.43–4878.72)	.18^b^
	Post	1058.73 (626.37–1482.51)	1094.05 (503.37–1399.96)		1079.86 (656.71–2389.23)	780.54 (251.26–1854.43)	
	p	.38^a^	.88^a^		.31^a^	1.35^a^	
AEA	Pre	201.95 (47.41–1518.88)	204.24 (69.82–396.43)	.29^b^	203.66 (18.33–1179.15)	204.53 (42.16–1645.95)	.68^b^
	Post	202.81 (47.31–1033.03)	203.36 (120.25–268.49)		226.59 (69.04–1111.20)	260.91 (80.53–1137.35)	
	p	.83^a^	.09^a^		.69^a^	.30^a^	
2-AG	Pre	91.10 (22.82–426.23)	102.61 (30.59–376.84)	.89^b^	67.55 (27.83–395.81)	171.79 (27.32–479.11)	.50^b^
	Post	91.69 (31.53–314.62)	102.05 (26.35–349.99)		139.58 (28.15–335.46)	96.79 (24.35–426.84)	
	p	.49^a^	.41^a^		.69^a^	.64^a^	

WOND: Lumbar disc herniation patients without neurological deficit; WND: Lumbar disc herniation patients with neurological deficit; TNF-α: Tumor necrosis factor alpha; IL: Interleukin; 1β: 1 beta, BE: Beta-endorphin; AEA: Anandamide, 2-AG: 2-Arachidonoylglycerol. Values are presented as median [IQR,25th–75th percentile].

a Wilcoxon test,

b Mann-Whitney U test.

**Table 6:** The descriptive and statistical comparisons of the clinical outcomes.

		WOND			WND		
		SE (n = 17)	GE (n = 17)	p	SE (n = 19)	GE (n = 16)	p
VAS	Pre	5.00 (0–10)	5.00 (3–10)	.56^b^	7.00 (0–10)	8 (0–10)	.06^b^
	Post	0.00 (0–2)	0.00 (0–5)		0.00 (0–7)	3.00 (0–7)	
	p	**.001** a	**<.001** a		**<.001** a	**.001** a	
ODI	Pre	40.00 (20–66)	44.00 (20–70)	.16^b^	42.00 (20–92)	48.00 (22–66)	.10^b^
	Post	4.00 (0–54)	22.00 (4–52)		12.00 (0–54)	26.00 (8–44)	
	p	**<.001** a	**.001** a		**<.001** a	**.001** a	
BDI	Pre	9.00 (3–31)	15.00 (4–26)	.19^b^	11.00 (5–26)	9.00 (5–24)	**.012** b
	Post	3.00 (0–18)	10.00 (3–22)		3.00 (0–19)	8.00 (0–23)	
	p	**.003** a	**<.001** a		**<.001** a	**.018** a	
BAI	Pre	9.00 (1–44)	9.00 (3–30)	**.024** b	7.00 (3–18)	8.00 (0–15)	**.049** b
	Post	2.00 (0–25)	9.00 (2–16)		2.00 (0–18)	5.00 (0–14)	
	p	**.001** a	**.017** a		**<.001** a	.107^a^	

WOND: Lumbar disc herniation patients without neurological deficit; WND: Lumbar disc herniation patients with neurological deficit; VAS: Visual Analog Scale; ODI: Oswestry Disability Index; BDI: Beck Depression Inventory; BAI: Beck Anxiety Inventory. Values in bold type were significant. Values are presented as median [IQR,25th-75th percentile].

a Wilcoxon test,

b Mann-Whitney U test.
