# Supplementary material for: Effects of lumbar stabilization exercises on serum biomarker levels and clinical outcomes in lumbar disc herniation: a subgroup analysis of a randomized controlled trial
Source: Turk J Med Sci. 2025 May 7;55(3):572–84. doi: 10.55730/1300-0144.6004 (PMC12270297; doi:10.55730/1300-0144.6004)
Supplement: Supplementary file 1 [file tjmed-55-03-572_Appendix.pdf]

Appendix

STABILIZATION EXERCISE PROGRAM

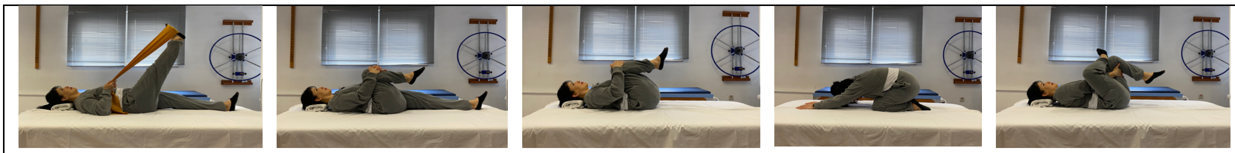

A) Stretching exercises

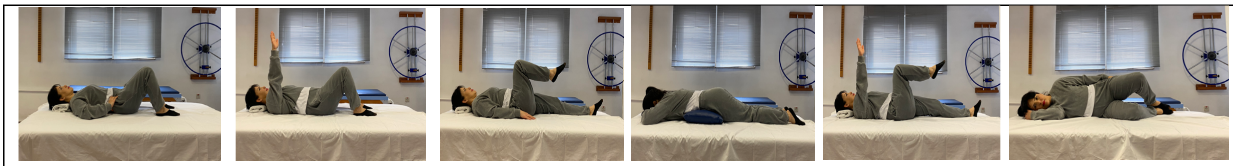

B) First week exercises

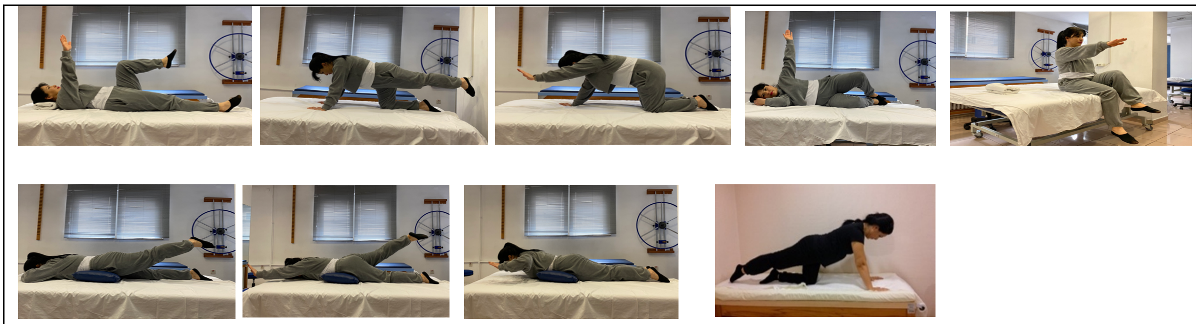

C) Second week exercises

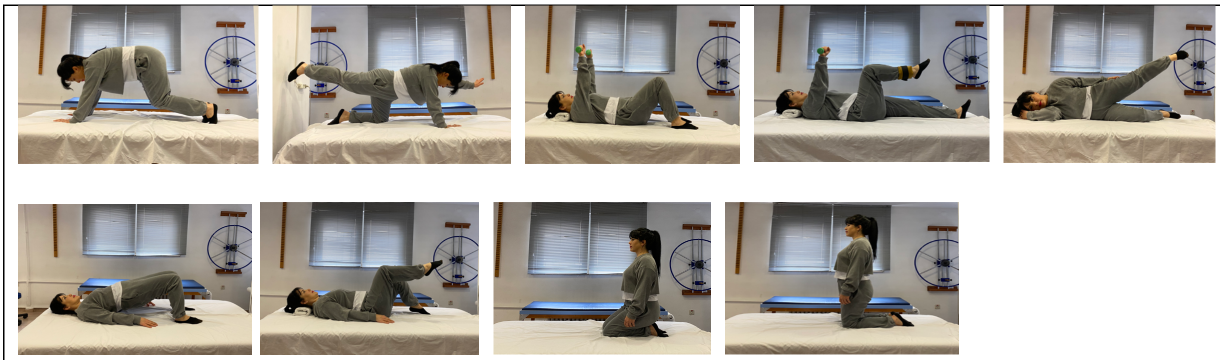

D) Third week exercises

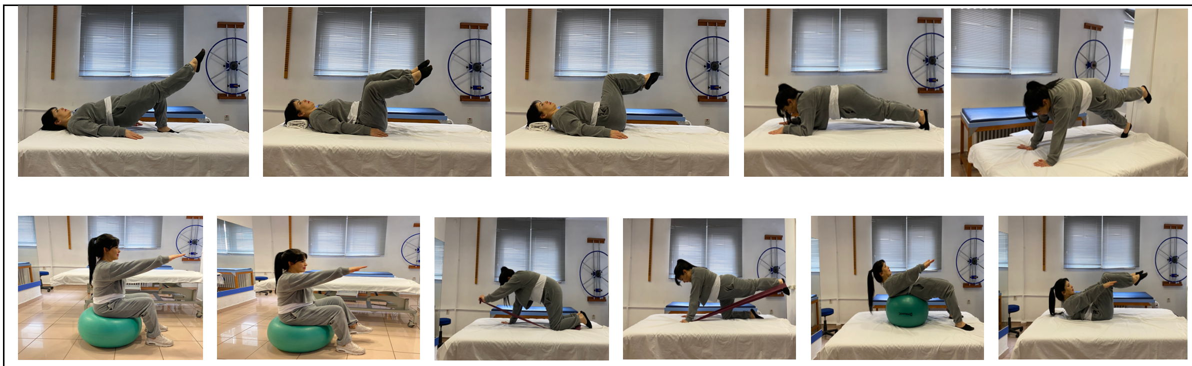

E) Fourth week exercises

STABILIZATION EXERCISE PROGRAM

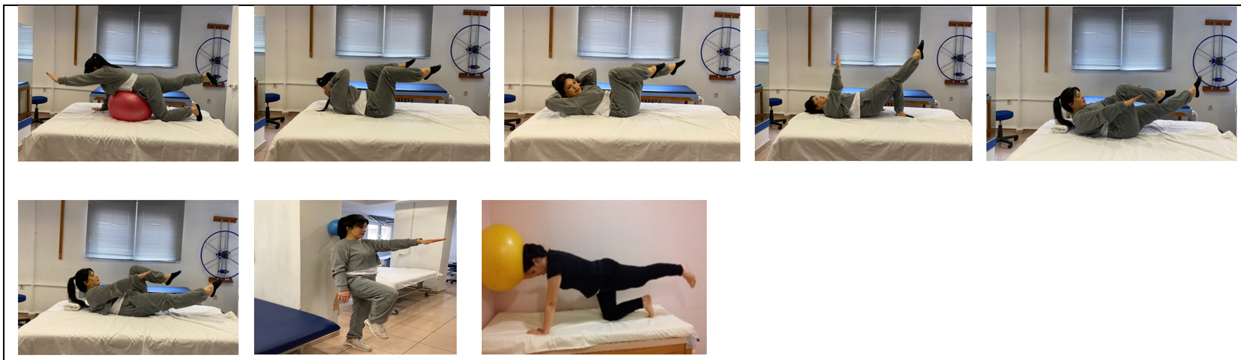

F) Fifth week exercises

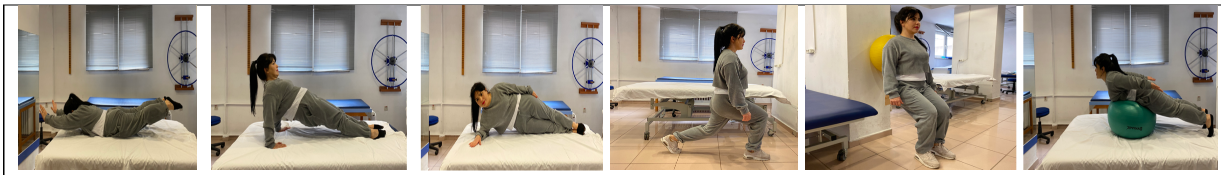

G) Sixth week exercises

GENERAL EXERCISE PROGRAM

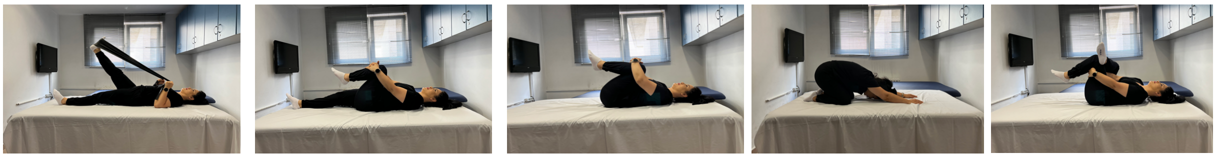

A) Stretching exercises

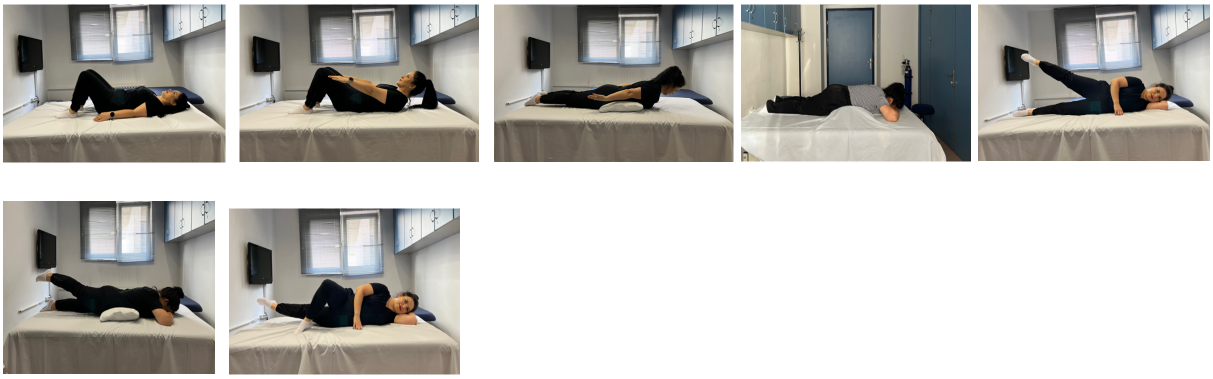

B) General exercises
